# RANK-RANKL Signaling in Cancer of the Uterine Cervix: A Review

**DOI:** 10.3390/ijms20092183

**Published:** 2019-05-02

**Authors:** Peter A. van Dam, Yannick Verhoeven, Julie Jacobs, An Wouters, Wiebren Tjalma, Filip Lardon, Tim Van den Wyngaert, Jonatan Dewulf, Evelien Smits, Cécile Colpaert, Hans Prenen, Marc Peeters, Martin Lammens, Xuan Bich Trinh

**Affiliations:** 1 Multidisciplinary Oncologic Center Antwerp (MOCA), Antwerp University Hospital, B2650 Edegem, Belgium; Yannick.Verhoeven@uantwerpen.be (Y.V.); Wiebren.Tjalma@uza.be (W.T.); Cecile.Colpaert@uza.be (C.C.); Hans.Prenen@uza.be (H.P.); Marc.Peeters@uza.be (M.P.); Martin.Lammens@uza.be (M.L.); XuanBich.Trinh@uza.be (X.B.T.); 2 Center for Oncological Research (CORE), University of Antwerp, B2610 Wilrijk, Belgium; julie.jacobs@uantwerpen.be (J.J.); an.wouters@uantwerpen.be (A.W.); filip.lardon@uantwerpen.be (F.L.); Tim.VandenWyngaert@uza.be (T.V.d.W.); jonatan.dewulf@uantwerpen.be (J.D.); Evelien.Smits@uza.be (E.S.); 3 Department of Nuclear Medicine, Antwerp University Hospital, B2650 Edegem, Belgium; 4 Department of Histopathology, Antwerp University Hospital, B2650 Edegem, Belgium; 5 Department of Histopathology, Gasthuiszusters Antwerpen (GZA) Hospitals, B2610 Wilrijk, Belgium

**Keywords:** RANK, RANKL, immunotherapy, checkpoint inhibition, cervical cancer, microenvironment

## Abstract

RANK ligand (RANKL) is a member of the tumor necrosis factor alpha superfamily of cytokines. It is the only known ligand binding to a membrane receptor named receptor activator of nuclear factor-kappa B (RANK), thereby triggering recruitment of tumor necrosis factor (TNF) receptor associated factor (TRAF) adaptor proteins and activation of downstream pathways. RANK/RANKL signaling is controlled by a decoy receptor called osteoprotegerin (OPG), but also has additional more complex levels of regulation. The existing literature on RANK/RANKL signaling in cervical cancer was reviewed, particularly focusing on the effects on the microenvironment. RANKL and RANK are frequently co-expressed in cervical cancer cells lines and in carcinoma of the uterine cervix. RANKL and OPG expression strongly increases during cervical cancer progression. RANKL is directly secreted by cervical cancer cells, which may be a mechanism they use to create an immune suppressive environment. RANKL induces expression of multiple activating cytokines by dendritic cells. High RANK mRNA levels and high immunohistochemical OPG expression are significantly correlated with high clinical stage, tumor grade, presence of lymph node metastases, and poor overall survival. Inhibition of RANKL signaling has a direct effect on tumor cell proliferation and behavior, but also alters the microenvironment. Abundant circumstantial evidence suggests that RANKL inhibition may (partially) reverse an immunosuppressive status. The use of denosumab, a monoclonal antibody directed to RANKL, as an immunomodulatory strategy is an attractive concept which should be further explored in combination with immune therapy in patients with cervical cancer.

## 1. Introduction

Although cervical cancer (CC) is traditionally regarded as a preventable disease, it continues to be a major health problem in many parts of the world. Globally it is the fourth most common cancer in women, with most cases diagnosed in an advanced stage [1]. A chronical infection with a human papilloma virus (HPV) plays a major etiological role in about 95% of invasive CCs [2]. Before developing an invasive carcinoma, cervical cells go through different precancerous stages, called cervical intraepithelial neoplasias (CINs). These are classified according to their grade of dysplasia: very mild to mild (CIN I), moderate (CIN II), and severe dysplasia to carcinoma-in-situ (CIN III). Most mild dysplastic lesions disappear spontaneously, but a significant proportion of the CIN III lesions may progress into invasive CC if left untreated [3]. These precancerous lesions have a likelihood of about 95% to be picked up in by screening programs using cytology and/or HPV testing on cervical smears [4]. They can be effectively eradicated by minimally invasive excision or ablation techniques, truly preventing CC. Primary prevention of CC is possible by vaccination against HPV. Patients diagnosed with early invasive CC are preferentially treated surgically: by a cone biopsy, hysterectomy, or radical hysterectomy with pelvic lymphadenectomy or sentinel node biopsy depending on their stage [5,6]. Curative concurrent chemotherapy combined with radiotherapy is the first treatment of choice for patients with more advanced CC (Federation International de Gynecologie et Obstetrie (FIGO) stages IB2 to IVA), but can also be used in earlier stages depending on comorbidity and patient preference [6]. Over the last decades metastatic disease was treated with platinum-based chemotherapy, but there has been little improvement in the efficacy of the systemic treatment [3]. The short-lived responses to chemotherapy seen in patients with advanced cervical cancer indicate that the disease is relatively chemorefractory and up to now most new targeted drugs provided no extra survival benefit [3,4]. The Gynecologic Oncology Group (GOG) 240 phase III trial showed that addition of bevacizumab to the current golden standard (combination of topotecan-paclitaxel or cisplatin-paclitaxel) in this population is associated with a moderate increased overall survival (16.8 months vs. 13.3 months) compared to the same cytostatic combinations without antiangiogenic medication. At 36 months the likelihood to be alive was about 10% in both patient groups illustrating the dismal prognosis of these patients [7]. Inoperable CC is likely to remain highly prevalent during the next decades as screening programs and vaccination campaigns against human papilloma virus (HPV) have suboptimal participation rates, are not completely effective, and are still unavailable in most countries [4]. 

It has been known for decades that HPV infection plays a crucial role in triggering most CCs, but only recently the regulatory networks involved have been unraveled [2]. Important driver pathways in CC carcinogenesis are associated with avian myelocytomatosis viral oncogne holog (MYC) signaling, cell cycle deregulation, TGFβ-signaling, mitogen-activated protein kinase (MAPK) signaling, and chromatin modeling [8]. In 2017, a comprehensive landmark study on invasive CC was conducted as part of The Cancer Genome Atlas (TCGA) project, aiming to identify relevant molecular events that drive tumorigenesis [9]. Integrated clustering identified keratin-low squamous, keratin-high squamous, and adenocarcinoma-rich clusters defined by different HPV and molecular features. Higher activation of p53, p63, p73, activator protein 1 (AP-1), MYC, hypoxia-inducible factor 1-alpha (HIF1A), fibroblast growth factor receptor 3 (FGFR3), and MAPK pathways were found to be distinguishing features of squamous cell carcinomas, while adenocarcinomas exhibited higher inferred activation of estrogen receptor alpha (ERα), forkhead box protein A1 (FOXA1), FOXA2, and FGFR1 signaling [9]. The authors could identify new frequently mutated genes (transforming growth factor beta 2 (TGFBR2), epiderma growth factor receptor 3 ERBB3, cysteine-aspartic acid protease 8 CASP8, SHKBP1 binding protein 1, and human leucocyte antigen A HLA-A, confirm previously reported significant gene mutations (phosphatidylinositol-4,5biphosphate 3 kinase *PIK3CA, KirstenRat sarcoma virus KRAS, phosphatase and tensin homolog PTEN, MAPK1, nuclear factor erythroid 2 like 2 NFE2L2, AT-rich intercative domain-containing protein 1A ARID1A, EP300, F-box/WD repeat-containg protein 7 FBXW7, HLA-B),* and detected striking apolipoprotein B mRNA editing enzyme catalytic polypeptide-like APOBEC mutagenesis patterns. They also discovered amplifications in the well-known checkpoint controlling immune targets programmed death ligand 1 (PD-L1, CD274) and PD-L2 (PDCD1LG2) in the keratin-low and -high squamous cancers, but not in the adenocarcinoma enriched group. In addition, amplifications in the long non-coding RNA of the breast cancer anti-estrogen resistance 4 (BCAR4) gene, which regulates the expression of perforin and granzyme A, were found [9]. This suggests that immunotherapeutic strategies may be active in a subset of cervical cancers. Preliminary studies confirm that responses are seen in a minority of patients with CC when they are treated with checkpoint inhibitors alone or in combination with chemo- or radiotherapy [10]. Recently, it has been suggested that RANK/RANKL inhibition has the potential to make tumors the more susceptible to checkpoint inhibition [11]. In the present paper, the literature (PubMed, Web of Science) on RANK/RANKL signaling in cervical cancer is reviewed up to March 2019. Furthermore, a translational window of opportunity study (DICER: denosumab in cervical cancer) starting in January 2019 is proposed investigating the effects of RANKL inhibition on the immune environment in patients with squamous carcinoma of the cervix.

## 2. The RANK/RANKL Signaling Network

The tumor necrosis factor (TNF) superfamily (TNFSF) is a superfamily of type II transmembrane proteins commonly containing the TNF homology domain [12]. This superfamily consists of more than 20 protein members, which can be released from the cell membrane by proteolytic cleavage by specific metalloproteinases to generate soluble cytokines [13]. The members of the TNFSF have in common that they interact with their cognate TNF receptor superfamily (TNFRSF) members [12,14]. Conserved residues provide specific inter subunit contacts. There is substantial crosstalk among related ligand receptor pairs and cytokines, and recent evidence suggests that signals can be generated from the receptor part (forward signaling) as well from the ligand part (reverse signaling) [12]. The TNFSF/TNFRSF axes are involved in regulating diverse biological processes, including embryogenesis, differentiation, proliferation, apoptosis, inflammation, and immune responses [14,15]. They provide a communication network that is essential for coordinating multiple cell types into an effective host defense system against pathogens and malignant cells [13].

**Figure 1:**
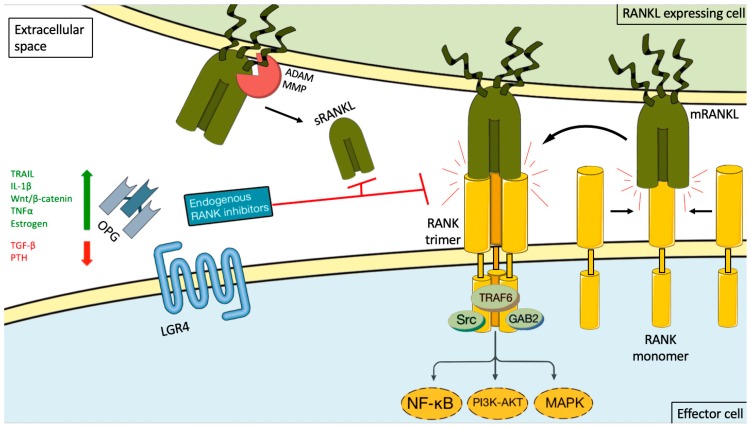
RANKL/RANK signaling and its endogenous inhibition. Binding between RANKL and RANK induces receptor trimerization, which triggers recruitment of TNF receptor associated (TRAF) adaptor proteins and activation of downstream signaling pathways (such as NF-κB, PI3K-AKT, and the MAP kinase cascade). This activity can be induced by either membrane-bound RANKL (mRANKL) or soluble RANKL (sRANKL). sRANKL is derived from the membrane-bound form through alternative splicing or proteolytic cleavage (for example, by matrix metalloproteinase (MMP) or disintegrin and metalloproteinase (ADAM) family members. RANK itself lacks kinase activity, and its signaling is initially mediated by adaptor molecules, such as TRAF proteins (including TRAF6), GRB-associated-binding protein 2 (GAB2), and sarcoma proto-oncogene tyrosine-kinase (SRC). The signaling cascade is controlled by a decoy receptor called osteoprotegerin (OPG) and leucine-rich repeat-containing G protein-coupled receptor 4 (LGR4). OPG expression can be upregulated by several factors such as TRAIL, IL-1β, Wnt/β catenin signaling, TNFα, and estrogen, and down regulated by TGF-β and PTH. The endogenous inhibitors bind both the soluble and membrane-bound RANKL forms, thereby preventing it from interacting with RANK. The green arrow stand for upregulation, the red arrow for inhibition and the black arrows for possible interactions and the curved black arrow for the result of interaction.

RANKL is a type II homotrimeric transmembrane protein bearing close homology to TNFSF members TNF-related apoptosis inducing ligand (TRAIL), Fas ligand (FasL, TNFSF6), and TNF-alpha [15]. It has three known isoforms that are expressed as membrane bound (RANKL1, RANKL2) or as soluble secreted proteins (RANKL3) by cleavage of the membranous forms by the metalloprotease–disintegrin TNF-alpha convertase (TACE), or by alternative splicing [15,16]. The RANKL is encoded by the TNFS11 gene in humans and is also named osteoprotegerin ligand (OPGL), TNF-related activation induced cytokine (TRANCE), or osteoclast differentiation factor (ODF) [16,17]. It is the only known ligand binding to a membrane receptor named receptor activator of nuclear factor-kappa B (RANK (TNFRSF11A) [17,18]. Binding between RANKL and RANK induces trimerization of the receptor, which triggers recruitment of TNF receptor associated factors (TRAF), adaptor proteins, and activation of downstream signaling pathways (such as NF-κB, RAC-alpha serine/threonine-proteine kinase (AKT), protein kinase B (PKB), c-Jun *N*-terminal kinase (JNK), and the MAPK cascade) [19]. RANK/RANKL signaling is controlled by a decoy receptor called osteoprotegerin (OPG, TBFRSF11B), which interacts with RANK and TRAIL (Figure 1). OPG is a soluble glycoprotein that can exist either as a 60 kDa monomer or as a 120 kDa dimer but has no transmembrane or cytoplasmatic domains. The dimerization of OPG increases the affinity of OPG to RANKL dramatically [20]. OPG expression can be upregulated by several factors such as TRAIL, Wnt/β catenin signaling, IL-1β, TNFα, and estrogen, and down regulated by TGF-β and parathyroid hormone (PTH) [17,21]. The final inhibitory effect of OPG on RANKL is dependent on its binding to these ligands [22,23]. Recently a new receptor for RANKL, LGR4 (leucine-rich repeat-containing G-protein coupled receptor 4), has been identified, which suppresses canonical RANK signaling by competing with RANK to bind RANKL [24]. Data are emerging that complex additional levels of regulation of the RANK/RANKL network exist [17,25,26].

## 3. Functional Role of RANK/RANKL Signaling

RANKL/RANK signaling is crucial for normal bone remodeling and homeostasis [27]. It regulates the formation of multinucleated osteoclasts from their monocyte-macrophage precursors as well as their activation and survival [27,28,29]. OPG protects the skeleton from excessive bone resorption by binding to RANKL, thereby preventing it from binding to RANK. [27,29]. The RANK pathway is deregulated in pathological processes like osteoporosis and cancer induced bone destruction [27]. It is also involved in chronical inflammatory diseases such as arthritis and inflammatory bowel disease [19]. RANK(L) has a functional role in normal tissues as it can be detected in epithelial and stromal components of the mammary gland, prostate, liver, thymus, and many other structures [23]. Studies in mice indicate that RANKL/RANK signaling is required for lymph node formation and mammary gland development [30,31,32]. RANK/RANKL and OPG knockout mice have a disrupted immune phenotype (e.g., impaired T or B cell development) [32]. 

It has been known for a long time that RANKL modulates the immune response by inducing lymphocyte differentiation, T-cell activation, and dendritic cell (DC) survival [33]. RANKL can be found in tumor infiltrating lymphocytes (TILs), B cells, immature dendritic cells, monocytes, and macrophages [34]. As RANK has a high homology with CD40 (another TNF receptor family member), it is reasonable to assume that RANK signaling initiates cellular responses similar to CD40 signaling by triggering common intracellular signaling pathways such as NFκB, p38, MAPK, c-JUN *N*-terminal kinase (c-JNK), and the extracellular signal-regulated kinases (ERK) [31,35,36]. Numerous studies have demonstrated that CD40 signaling induces the maturation, activation and survival of DCs [31]. Dendritic cells process and present antigens, thereby priming and activating T cells during the immune response. RANKL induces expression of multiple activating cytokines by DCs, including IL-1, IL-6, IL-12, and IL-15 [37]. The RANKL signal can alter the function of dendritic cells, increasing the number of Foxp3-positive regulatory T cells (Tregs). These Tregs are a CD4^+^ helper T cell subset, critical for suppressing autoimmune responses in the body [38]. RANKL signaling can also enhance DC survival via induction of the antiapoptotic protein Bcl-xL (B-cell lymphoma-extra large) [39]. Tumor associated macrophages (TAMs) are important components of the tumor environment and are crucial to maintain a status of local immunosuppression surrounding the tumor [40,41,42]. They express immune checkpoint modulators (such as PD-L1) that directly suppress activated T cells and produce various chemokines that attract other immunosuppressive cells such as Tregs, myeloid-derived suppressor cells (MDSCs), and type 2 helper (Th2) T cells [43]. As RANKL is mainly produced by Treg lymphocytes, a vicious cycle can be established in conjunction with the TAMS that mainly express RANK. In this context RANKL acts as a chemoattractant for these cells [40]. Depending on their M2 (alternatively activated) or M1 (activated) phenotype, TAMs produce matrix metalloproteinases (MMPs), which play important roles in tissue remodeling (e.g., physiologic tissue repair, but also metastasis) [17,19,44]. Treg cells have also been shown to stimulate mammary cancer metastasis directly through RANKL-RANK signaling or by enhancing the tumor permissive properties of macrophages [40]. Tumor cells can produce or entrap OPG by the proteoglycans and glycosaminoglycans of the extracellular matrix, creating a microenvironment that facilitates the expansion of the tumor cells [17,45]. Moreover, exposure of natural killer cells (NK) to tumor-derived RANKL upregulates RANK expression of the NKs and impairs their antitumor response, suggesting yet another possible positive feedback loop that facilitates the evasion of tumor cells from NK cell-mediated immune surveillance [46]. 

The impact of RANK-RANKL signaling on the tumor behavior and the immune environment is context specific. In many situations the RANK network seems to play a crucial role in creating an immunosuppressive microenvironment that promotes tumor progression. The pivotal role of RANK/RANKL signaling in bone metastasis has been well documented [47,48,49,50]. The RANK-mediated signal network can drive epithelial to mesenchymal transition (EMT) in cancer cells, promote osteomimicry, induce stem cell-like cell phenotypes, and give cancer cells the ability to home to bone [17,42,45,46,47]. RANK expression in primary tumors was a predictive marker the occurrence of bone metastasis in a large population of breast cancer patients [51]. However, RANK signaling seems to be important in the biology of many tumor types beyond bone metastasis [11,17,23,34]. RANK and RANKL expressing tumor cells are commonly found in the tumor microenvironment [23,45,49]. The RANKL/RANK pathway is often overexpressed in cancers of the breast, prostate, endometrium, cervix, stomach, esophagus, bladder, and thyroid and this is correlated with poor prognosis [11,17,23,34,50,51,52,53,54,55,56,57,58]. It has been shown that RANK/RANKL signaling can also promote the initial stages of cancer development by inducing stemness and EMT in vitro and in vivo [11,59,60,61,62,63]. RANKL has been detected in endothelial cells and has also been implicated in angiogenesis [64]. 

## 4. RANK-Mediated Signaling and Cervical Cancer

Until recently little was known on the role of the RANKL-RANK signaling network in cervical cancer. In 2015, Shang et al. showed that RANK and RANKL were co-expressed in carcinoma of the uterine cervix. They assessed the expression of RANKL and RANK in the cervical cancer cell lines SiHa and HeLa and in twelve cervical cancer tissues. Stronger membrane staining for RANKL and RANK was detected in the cell lines and in cancer cells compared to precancerous cells [65]. Other groups confirmed that cytoplasmatic immunoreactivity for RANKL is weak in normal cervical tissues and became significantly higher during neoplastic transition from cervical intraepithelial neoplasia (CIN) I to CIN III and invasive CC [66,67]. In a series of 110 patients with invasive CC, the expression of RANK mRNA was significantly correlated with tumor pathological grade, clinical stage, depth of invasion, and lymph node metastasis. RANK is a direct binding target of KLF5 (Krüppel-like factor 5), a zinc finger containing protein [67]. In vitro experiments showed that KLF5 promotes proliferation, migration, and invasion of HeLa cells by up-regulating the transcription of RANK. Tumor necrosis factor (TNF)-α induced KLF5 expression by activating the p38 signaling pathway [67]. High KLF5 or RANK expression increased the risk of death in patients with squamous CC [67]. It was previously demonstrated that hypoxia can upregulate the expression of RANK and RANKL, and increases RANKL-induced cell migration via the PI3K/AKT-hypoxia inducible factor-1α (HIF-1α) pathway [68]. Therefore, these inflammatory factors and local hypoxia may contribute to a high level of RANKL/RANK in cervical cancer cells.

Cervical cancer cells secrete soluble RANKL (soRANKL) [66]. Blocking RANKL/RANK interaction by recombinant OPG or anti-RANKL neutralizing antibodies significantly decreases proliferation and increases apoptosis, suggesting that the stimulatory effect of RANKL on the growth of cervical cancer cells may be mainly dependent on membrane RANKL/RANK interaction [66]. In an immunodeficient mouse model, RANK-knockdown suppresses the metastatic potential of Hela cells. Antibodies against RANKL reduce the secretion of IL-8 by SiHa and Hela cells, thereby inducing apoptosis and reducing proliferation. On the other hand, recombinant IL 8 has the opposite effect [66]. This indicates that RANKL may stimulate cervical cancer growth by activation of the IL-8 pathway. Jia et al. recently showed that the expression of IL-8 was significantly higher in cervical carcinoma samples compared to normal controls and that migration and proliferation of Hela cells increased after treatment with IL-8 [69]. Survival of cervical cancer patients with high intratumoral IL-8 expression is significantly worse compared to patients with low IL-8 in their tumors [70]. Strikingly, these authors identified colocalization between IL8 and CD66 (tumor associated macrophage staining), and a correlation with microvessel density counts, vascular endothelial growth factor VEGF, fibroblast growth factor FGF, and platelet derived growth factor PDGF levels. This suggests that IL-8 is likely to be involved in angiogenic growth and creating an immune suppressive environment. In a xenograft mouse model using cervical cancer cells with a high IL-8 production (CaSki cells) treatment with an antibody against IL-8 resulted reduced tumor growth and lymph node metastases and prolonged survival significantly [71].

Only a minority of genital human papillomavirus (HPV) infections progress into (pre)neoplastic lesions [2]. Human papilloma virus can use multiple mechanisms to evade the immune surveillance, to alter cell cycle control, and to facilitate the accumulation of DNA damage and malignant transformation of the cervical keratinocytes [36]. In an elegant set of experiments, Demoulin et al. identified RANK/RANKL signaling as a likely candidate driving the tolerogenic alterations in DCs in the genital (pre)neoplastic microenvironment [66]. They showed that squamous cervical and vulvar cancer cells (CVCC) secrete factor(s) that can affect the maturation and function of dendritic cells (DC) via RANK pathway signaling. Dendritic cells incubated with recombinant RANKL had reduced maturation characteristics, lower IL-12 and higher IL-10 expression, suggestive for an immunosuppressive status. OPG can inhibit this effect and restore DC functions. Dendritic cells cocultured with CVCCs induce FoxP3^+^ suppressive Treg cells from naive CD4^+^ T cells. Therefore, RANKL inhibition seems to be an attractive approach to increase the immunogenicity of cervical cancer. Garcia Paz et al. showed in a HPV16-positive tumor bearing syngeneic mouse model that treatment with the IL-12 gene increased the intratumoral expression of IL-12, IL-2, and gamma-interferon (being immune stimulatory) and reduced the expression of TGF-β1, IL4, and IL10 (known to be immunosuppressive) [72]. IL-12 gene transfer activated the immune response, inhibited tumor growth and prolonged survival of the mice. This proves the potential therapeutic concept of altering the immune response in cervical cancer.

Weak immunostaining for OPG could be detected in normal and metaplastic exocervix, while OPG expression was significantly higher in (pre)cancerous lesions regardless of their grade [73]. In a series of 218 patients with CC treated with primary surgery, 42% of patients with squamous carcinomas, and 49% of patients with adenocarcinomas had high OPG expression levels. High OPG expression was correlated with high FIGO stage, tumor grade, presence of lymph node metastases, and poor overall survival. It proved to be an independent prognostic factor in multivariate analysis [73]. Osteoprotegerin was amongst six proteins having significantly different levels in CC patients compared to controls and CIN. This confirms that OPG may be involved in cervical cancer carcinogenesis and has the potential to be used as a biomarker [74].

In an in silico study comparing stromal and immune response related genes in normal cervix versus cervical carcinoma (unpublished data), we identified TMPRSS11D (encoding a serine-like trypsine protease) and CLCA2 (human chloride channel accessory-2 gene) among the top ten differentially expressed genes [8]. TMPRSS11D directly interacts with OPG [75]. In mice the ion channel CLCA2 is significantly induced by RANKL stimulation [76]. CLCA2 encodes a member of the calcium-activated chloride channel regulator (CLCR) family of proteins that regulates the transport of chloride across the plasma membrane. Expression of CLCA2 is upregulated by the tumor suppressor protein p53 in response to DNA damage and this has a significant effect on cell migration and invasion [77]. 

The above data indicate that RANK/RANKL signaling is an important player involved in the initiation, progression, proliferation, migration, and invasion of CC, and in modulating the immune environment. Theoretically RANKL inhibition is an intriguing target for CC for two main reasons. First, the high levels of expression of RANK and its ligand in a large proportion of CC’s, the in vitro effects of RANKL inhibition on proliferation and the correlation with prognosis of high RANKL expression make it likely that there may be a direct antiproliferative effect on the tumor cells and a reduction of the metastatic potential. Secondly there is abundant circumstantial evidence suggesting that the effects on the microenvironment may (partially) reverse an immunosuppressive status [78]. As CC is a chronic inflammatory disease with a high mutational load combining RANKL inhibition with immunotherapy is an attractive therapeutic option which should be further explored [79].

## 5. The Effect of RANK Signaling Inhibition on Non-Bone Tumor Growth in Humans

Denosumab is a fully human monoclonal antibody that binds RANKL, thereby blocking its interaction with RANK [78,80]. The drug is approved for the treatment of osteoporosis and giant cell tumor of the bone, and for the prevention and treatment of skeletal complications caused by bone metastases and lytic bone lesions in multiple myeloma [81]. Denosumab has a well-known and acceptable toxicity profile, alone or in combination with other treatment modalities such as cytotoxic medication, targeted drugs, and radiotherapy [78,81]. It remains unclear whether RANK/RANKL inhibition with denosumab in patients with cancer has any effect beyond the bone. In an post hoc analysis of patients with non-small cell lung carcinoma (NSCLC) included in a phase III randomized trial comparing zoledronic acid versus denosumab, a survival benefit was observed (hazard raio (HR) 0.80; 95% onfidence interval (CI) 0.67–0.95, *p* = 0.01) for the patients treated in the denosumab arm [82]. As no difference was seen in delay of bone events in both groups, and the beneficial effect of denosumab could also be observed in patients with visceral metastasis it is tempting to suggest that RANK/RANKL inhibition induces a survival benefit that is not related to a better control of bone disease. However, the recent prospective “Survival imProvement in Lung cancERNduced by DenOsUmab therapy” (SPLENDOUR) trial could not show any improvement in overall survival (OS) or progression free survival (PFS) by adding denosumab to standard first-line therapy in patients with metastatic NSCLC [83]. 

The effect of adjuvant denosumab in women with breast cancer was recently studied in two large multicenter prospective randomized trials [84,85]. In the Austrian Breast and Colorectal Cancer Study Group (ABCSG)—18 study (comparing placebo or denosumab 60 mg subcutaneously every six months for five years in 3425 postmenopausal patients with hormone sensitive early breast cancer treated with an aromatase inhibitor) it was shown that disease free survival was significantly better in the denosumab group [84]. In the Denosumab for Early Breast Cancer (DCARE) study (assessing 4509 high risk early breast cancer patients treated with standard therapy either with or without denosumab 120 mg subcutaneously (SC) every month for six months, then every three months up to five years) no improvement in bone metastasis free survival, disease-free, or overall survival was reported, even though there was an improvement in time to bone metastasis as the site of first recurrence in the denosumab group [85]. It is important to mention that most (95.9%) of these patients had received taxane or anthracycline based chemotherapy. Intriguingly, this lack of effect was also apparent in the subgroup analysis of postmenopausal women, conflicting with the results of the Adjuvant Zoledronic Acid to Reduce Recurrence (AZURE) trial, which had a similar study design but used zoledronic acid instead [86]. This raises the hypothesis that chemotherapy may reduce some of the tumor suppressive effects of RANK/RANKL inhibition in the cancer microenvironment. Other explanations may be the differences in molecular tumor characteristics of these patient populations, or effects of the menopause and endocrine treatment on the tumor behavior. It is clear that more research is warranted to elucidate the effect of denosumab on tumor growth. In the “Denosumab in Young Patients with Primary Breast Cancer” (D-BEYOND) trial, the biological effects of two neoadjuvant injections of 120 mg denosumab (one week apart) in 27 patients with premenopausal primary breast cancer were evaluated [87]. The authors concluded that two weeks of RANKL inhibition did not have an effect on the tumor proliferation rate but significantly increased the number of tumor infiltrating lymphocytes (TILs) in the tumor environment, making them theoretically more susceptible for immune therapy. Recently, there some additional evidence emerged that RANK/RANKL inhibition may have a role as immune modulator [11,17,88,89,90,91,92,93]. In preclinical studies RANKL blockade improves the efficacy of anti cytotoxic T-lymphocyte-associated protein 4 (anti-CTLA4) targeted antibodies in solid tumor models of metastasis [88,89,92]. Bakhru et al. showed that antibodies blocking RANKL and CTLA4 cooperate to increase the presence of tumor infiltrating CD4+ T cells expressing cytolytic markers, thereby improving antimelanoma immunity [93]. Addition of RANKL blockade to anti-PD1 and anti CTLA4 resulted in superior tumor responses and was most effective if RANKL inhibition was given concurrent or following check-point blockade [93]. This triple combination therapy improved T cell effector function in tumor bearing mice by increasing the proportion of tumor infiltrating CD4+ and CD8+ T cells that can produce both interferon gamma and TNF. In a retrospective analysis, Afzal and Shirai found a synergistic effect of immune checkpoint inhibitors and denosumab in metastatic melanoma patients [89]. Further prospective studies are ongoing to explore the combination of denosumab with checkpoint inhibitor immunotherapy. In the phase 1b-2 CHARLI study, the Australia and New Zealand Melanoma Trials group (NCT03161756) compares nivolumab-denosumab versus ipilimunab-nivolumab-denosumab in patients with unresectable stage III/IV melanoma. The Australia and New Zealand Urogenital and Prostate Cancer Trial Group currently performs a phase 2 study “Denosumab and Pembrolizumab in Clear Cell Renal carcinoma “(KEYPAD, NCT03280667) trial of denosumab and the PD-1 inhibitor pembrolizumab in patients with renal cell carcinoma [78]. Clinical trials utilizing systemic administration of TNF-alpha and some other members of the TNFSF superfamily have resulted in an unacceptable level of toxicities, blocking their development up to now [12]. 

## 6. A Window of Opportunity Neoadjuvant Phase II Study Assessing the Therapeutic Potential of RANK/RANKL Signaling Inhibition in Patients with Primary and Recurrent Squamous Carcinoma of the Uterine Cervix (ISS 20177041)

**Figure 2:**
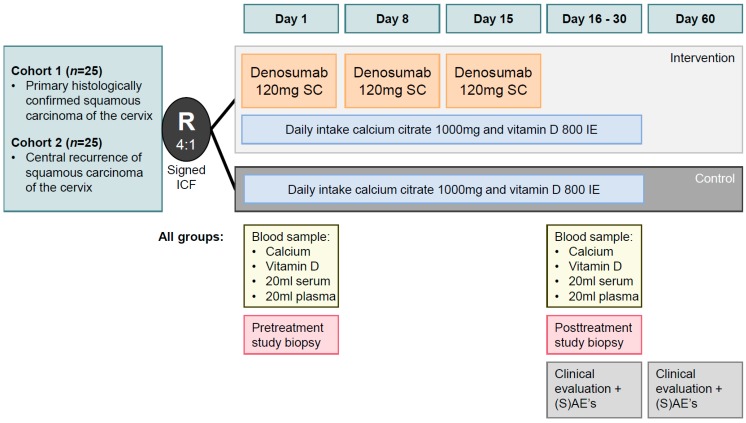
Outline of the DICER protocol: A window of opportunity neoadjuvant phase II study assessing the therapeutic potential of RANK/RANKL signaling inhibition in patients with primary and recurrent squamous carcinoma of the uterine cervix (ISS 20177041).

In vitro and animal studies cannot sufficiently mimic the effects of inhibition of RANK(L) on the immune environment in the human body. Encouraged by the preliminary results of the investigator sponsored proof of concept BRCA-D (Denosumab in BRCA positive breast cancer) study in BRCA positive breast cancer and the D-BEYOND study in luminal B breast cancer patients, we started a window-of-opportunity trial (DICER: Denosumab In Cervical Cancer) in January 2019 in patients with primary or recurrent squamous carcinoma of the uterine cervix [68,83]. It was decided to limit the study to squamous CC as this made the study population more homogeneous. The TCGA analysis showed that squamous and adenocarcinomas of the cervix have clearly distinct molecular and HPV infection related features with alterations to check-point controlling genes, hypermethylation, and downregulation of miR-200a/b mainly restricted to squamous carcinomas [6]. In this prospective multicenter neoadjuvant translational phase II study consecutive patients with primary FIGO stage IB–stage IIB squamous CC, and consecutive patients with a central recurrence of squamous CC will be randomized (4:1) in a parallel group design. Ethical approval has been obtained. The time interval between previous treatment and the central recurrence should be at least six months in the patients with recurrent disease in order to allow for a wash-out period of prior treatment. Prior treatment with denosumab or biphosphonates is not allowed. After informed consent the 20 patients with primary CC and 20 patients with recurrent CC in the active treatment cohort will have a first biopsy (using a Spirotome Single use Cervicore 10 Gauge, Medinvents, Belgium) at day 1, followed a by a subcutaneous injection of denosumab at days 1, 8, and 15 (Figure 2). The control arm will consist of five patients with primary and five patients with recurrent CC who will be treated with calcium carbonate 1000 mg and Vit D 800 International Units (IU) a day orally, without denosumab during 16–30 days, having similar biopsies as the patients who have active treatment. A second biopsy will be taken in a window between day 16–30 (using a Spirotome) in both groups. The primary objectives of this trial are to study the biological effects of two weeks neoadjuvant denosumab as detailed below, and to establish the safety and tolerability of this neoadjuvant treatment schedule. In particular the day 1 and 16–30 samples will be compared focusing on assessing effect on RANK signaling (immunohistochemical staining for RANKL, RANK, OPG, and RNA sequencing), assessing proliferation (scoring mitotic activity index on Hematoxylin and eosine stained (H&E) slides and immunohistochemical staining for Ki67 and RNA sequencing) assessing effect on angiogenesis (immunohistochemical staining CD31 and scoring microvessel density and RNA sequencing), assessing the effects on immune response (pancytokeratin-CD8 staining and PD-L1 immunohistochemical staining, RNA sequencing, flow cytometry for immune response markers). In addition, the side effects related to the treatment with denosumab will be registered prospectively during the neoadjuvant treatment period and also the first 30 days after treatment. The investigators will ascertain that the neoadjuvant study does not cause any delay of the planned treatment of cervical cancer, but it is mandatory that the second biopsy is taken before definitive treatment is started.

## 7. Conclusions

Identification of the factors involved in the cross talk between tumor cells, stroma, and immune cell subsets may provide opportunities to improve cancer treatment. RANKL signaling is likely to be a crucial player in cervical cancer. Theoretically RANKL inhibition can have a direct effect on tumor cell proliferation and behavior, but is also likely to alter the microenvironment surrounding the tumor cells, particularly the immune infiltrate. Further research is important to increase our knowledge in this field and particularly neoadjuvant trials are attractive to study these effects in vivo [5]. The use of denosumab as an immunomodulatory strategy is an attractive concept which should be further explored in combination with other treatment modalities.
